# Genetic inference of the etiological crosstalk between primary glaucomas and retinal vascular occlusions

**DOI:** 10.1016/j.aopr.2026.01.005

**Published:** 2026-02-13

**Authors:** Chuchu Wang, Tianyi Zhou, Yi Yu, Kaixuan Tang, Xinyi Luo, Wenming Shi, Xiaotong Yu, Jianfeng Luo, Yuchen Cai, Xinmin Lu, Yifan Zhou

**Affiliations:** a Department of Biostatistics, School of Public Health, Fudan University, Shanghai, China; b NHC Key Laboratory of Health Technology Assessment, Fudan University, Shanghai, China; c Key Laboratory of Public Health Safety of Ministry of Education, Fudan University, Shanghai, China; d Department of Ophthalmology, Shanghai Ninth People's Hospital, Shanghai Jiao Tong University School of Medicine, Shanghai, China; e Department of Ophthalmology, The First Affiliated Hospital of Anhui Medical University, Hefei, China; f Department of Ophthalmology, Shanghai Putuo Hospital, Shanghai University of Traditional Chinese Medicine, Shanghai, China; g School of Health and Rehabilitation Sciences, University of Pittsburgh, Pittsburgh, PA, USA; h Li Ka Shing Faculty of Medicine, The University of Hong Kong, Hong Kong, China; i Center of Basic Medical Research, Institute of Medical Innovation and Research, Peking University Third Hospital, Beijing, China; j Department of Plastic and Reconstructive Surgery, Shanghai Ninth People's Hospital, Shanghai Jiao Tong University School of Medicine, Shanghai, China; k Department of Ophthalmology, Shanghai Sixth People's Hospital, Shanghai Jiao Tong University School of Medicine, Shanghai, China; l Department of Ophthalmology, Shanghai Tenth People's Hospital, School of Medicine, Tongji University, Shanghai, China

**Keywords:** Primary glaucoma, Retinal vascular occlusion, Mendelian randomization, Mediation analysis, Blood pressure

## Abstract

**Purposes:**

The etiological connection between types of primary glaucoma and the risk of retinal vascular occlusions remains elusive. We conducted a two-sample bidirectional Mendelian Randomization (MR) study with mediation analysis to elucidate causal genetic relationships and investigate potential mediating pathways.

**Methods:**

Genetic instruments of primary glaucomas and retinal vascular occlusions were derived from the UK Biobank and the Finngen, respectively. A two-sample MR and reverse MR were conducted to elucidate directional and causal relationships. Ocular and systemic risk factors, including glaucomatous endophenotypes, retinal vasculature indexes, diabetes, hypertension, blood pressure (BP), cardiovascular diseases, and stroke, were also obtained from independent genome-wide association studies (GWAS) studies to investigate the mediating effects. Inverse variance weighting (IVW) was the primary analytical tool used to identify causality, and the results were verified through comprehensive and sensitivity tests for pleiotropy, heterogeneity, and stability.

**Results:**

Genetically predicted primary open-angle glaucoma (POAG) may elevate retinal vein occlusion (RVO, central or branch) risk (odds ratio (OR)=1.103, 95% confidence intervals (CI): 1.008-1.208, *P*=0.032). In the reverse direction, RVO (central or branch) was associated with an OR of 1.258 for primary angle-closure glaucoma (PACG); however, this association did not reach statistical significance (95% CI: 0.996-1.589, *P*=0.054). Mediation analysis suggested that 7.4% and 4.9% of the total effect of POAG on RVO (central or branch) risk were mediated in part through systolic and diastolic BP, respectively. These primary results remained robust through a variety of sensitivity tests.

**Conclusions:**

Our findings suggest that POAG may increase the risk of RVO, and this causal effect may be mediated in part through systolic and diastolic BP.

## 1 Introduction

Retinal vascular accidents in patients with primary glaucomas and the prevalence or incidence of types of primary glaucoma in patients with retinal vascular occlusions have gradually developed into a heated topic under discussion over the past century^1^, ^2^, ^3^. Quite a few studies have reported a higher prevalence/incidence of primary open-angle glaucoma (POAG) or primary angle-closure glaucoma (PACG) in patients with retinal vein occlusion (RVO) in the follow-up periods than in the general population^1^, ^2^, ^3^, ^4^, ^5^, and vice versa, prior POAG and PACG encroach on a large proportion of patients with newly diagnosed RVO.^6^ Moreover, glaucoma has also been recently reported as a risk factor for retinal artery occlusion (RAO).^7^ As such, the epidemiological relationships and etiological connections between primary glaucoma and retinal vascular occlusions warrant a thorough examination.

Elevated intraocular pressure (IOP), thinning of retinal nerve fiber layer (RNFL), enlarged optic cup, and retinal vascular distortion in the glaucomatous conditions may jeopardize the supporting structure of the inner retina, contribute to the retinal artery collapsing over the crossing vein, elevate retinal vein pressure, and reduce adequate perfusion to the inner retina and optic disc, which eventually leads to blood stasis and retinal vascular occlusion pathogenesis.^8^^,^^9^ Therefore, some researchers consider primary glaucoma to be the primary event in the glaucoma-retinal vascular occlusion interplay.^10^ On the other hand, the disruption of inner retinal circulation in RVO may, in reverse, lead to RNFL thinning, which may not only interfere with the diagnosis of glaucoma according to optic nerve head analysis but also signify impaired peripapillary perfusion.^11^ Patients undertaking standard treatment for glaucoma (especially POAG and normal tension glaucoma) may still encounter exacerbated glaucomatous progression owing to low ocular blood flow, impaired peripapillary perfusion, dysregulated 24 h mean arterial pressure, and other vasculature-related risk factors^12^, ^13^, ^14^, ^15^. RNFL thinning and peripapillary microvascular perfusion impairment in glaucomatous conditions may also occur in fellow eyes of RVO patients.^16^ Additionally, glaucoma development has been reported in the fellow eyes of patients with unilateral non-arteriovenous-crossing RVO.^1^ As such, bidirectional pathogenesis for types of primary glaucoma and ocular vascular occlusions has been noted in the existing literature, and a complete consensus has not been reached on the correlation between primary glaucomas and retinal vascular occlusions. The question of "Which comes first" between types of primary glaucoma and retinal vascular occlusions remains elusive to us.^2^

Previous reports on this topic have relied on observational studies of retrospective or prospective designs with a limited number of observed objects, which encounter the natural limitations of establishing causality and confounder bias. Mendelian randomization (MR) is an analytical method that utilizes genetic variations as randomized tools to investigate the causal effects of exposure on disease outcomes.^17^ To extend our understanding of the correlations between primary glaucomas and retinal vascular occlusions, the present study employed a bidirectional two-sample MR approach to explore the genetic insights into causal relationships between types of primary glaucoma (POAG/PACG) and retinal vascular occlusions (RVO/RAO). Moreover, we enrolled glaucomatous endophenotypes, some of which are meanwhile retinal construction abnormalities in retinal vascular occlusion conditions as well, including IOP, RNFL thickness, ganglion cell inner plexiform layer (GCIPL) thickness, vertical cup-disc ratio, and comprehensive risk factors for both primary glaucomas and retinal vascular occlusions, such as diabetes and blood pressure (BP), to make proper mediation analyses among causal relationships.

## 2 Materials and methods

### 2.1 Overall survey design

**Fig. 1:**
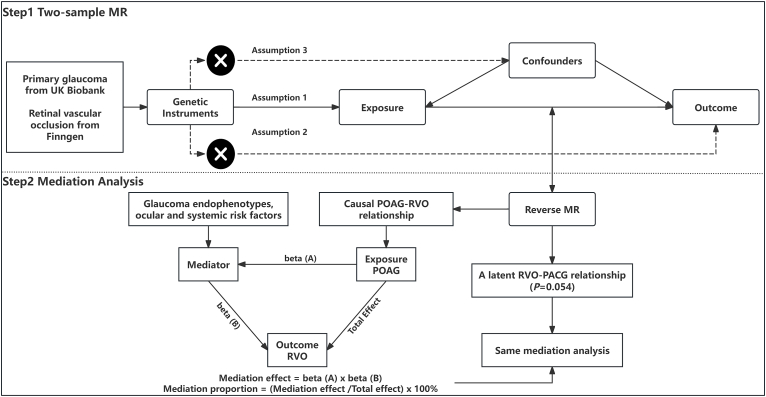
Overview of the study design and MR-based mediation framework. Genetic IVs are used to assess causal relationships, subject to three core assumptions regarding the use of IVs. After performing bidirectional UVMR among exposures, mediators, and outcomes, a two-step MR approach is conducted to explore potential mediation pathways.

The overarching design of the current study is depicted in Fig. 1. This study was conducted in accordance with the STROBE-MR guidelines.^17^ We utilized summary data from genome-wide association studies (GWAS) to evaluate the causal relationships between types of primary glaucoma (POAG/PACG) and retinal vascular occlusions (RVO/branch retinal artery occlusion (BRAO)/central retinal artery occlusion (CRAO)) using two-sample Mendelian randomization (TSMR). The validity of the MR analysis relied on three fundamental assumptions^18^: (1) the genetic variants, termed single nucleotide polymorphisms (SNPs), used as instrumental variables (IVs) are not linked to any confounder; (2) the IVs have a strong correlation with the exposure; (3) the IVs influence the outcome only through the exposure (Fig. 1). Next, sensitivity analyses, including the MR-Egger intercept test, Cochran's *Q* test, and leave-one-out sensitivity analysis, were used to evaluate the pleiotropy, heterogeneity, and stability of the univariable two-sample Mendelian randomization (UVMR) results. We first set primary glaucomas as the exposure, so a reverse MR analysis was performed not only to verify the direction of filtered causal links but also to explore causal relationships when retinal vascular occlusions were set as exposures. Then, we further investigated the mediating roles of glaucomatous endophenotypes, including IOP, RNFL/GCIPL thickness, vertical cup-disc ratio, and comprehensive risk factors such as retinal vasculature indexes (retinal arteriolar/venular tortuosity, retinal arterial/venular distance factor tortuosity, retinal arteriolar/venular width, and retinal vascular changes and abnormalities), type1/2 diabetes, hypertension, systolic and diastolic BP, cardiovascular diseases, and stroke among filtered causal links. Since all data are publicly accessible, and appropriate participant consent and ethical approval were obtained from the original studies, ethical approval from an institutional review board was unnecessary for the current investigation.

### 2.2 Data sources

Using the IEU OpenGWAS Project and the GWAS database (https://gwas.mrcieu.ac.uk/datasets/; https://www.ebi.ac.uk/gwas/downloads/summary-statistics, last visit for update: August 2024), summary data were obtained for analysis. The primary glaucoma data come from the UK Biobank, and the retinal vascular occlusions come from the most updated Finngen data (R11, CRAO; R9, BRAO, and RVO). Since the datasets of exposures and outcomes were derived from UKB and Finngen, respectively, there should be less concern about sample overlap. The enrollment of mediators relied on the existing MR studies on genetic prediction of risk factors for either primary glaucoma or retinal vascular occlusions. Mediator data were primarily obtained from the European primary population's summarized data on the IEU OpenGWAS Project, to minimize bias due to population heterogeneity (a detailed description of GWAS ID, sample size, and SNP number is provided in Table 1).

**Table 1:** Data source.

Items	GWAS ID	Year	Sample size	SNP number
Exposure/Outcome				
Primary open-angle glaucoma (PheCode 365.11)	GCST90043781	2021	654 cases/455694 controls	11842647
Primary angle-closure glaucoma (PheCode 365.2)	GCST90043782	2021	591 cases/455757 controls	11842647
Retinal vein occlusion (central or branch)	https://www.finngen.fi/en	2023	775 cases/308633 controls	20168406
Retinal artery occlusion (branch)		2023	201 cases/308633 controls	20168380
Central retinal artery occlusion		2024	677 cases/413784 controls	21306207
**Mediator**				
Ganglion cell inner plexiform layer thickness	GCST90014267	2021	31434	9121075
Retinal nerve fibre layer thickness	GCST90014266	2021	31434	9121075
Vertical cup-disc ratio	GCST004075	2017	23899	11149520
Intraocular pressure	GCST004074	2017	29578	11458861
Retinal arteriolar tortuosity	GCST90270397	2023	52798 European as discovery sample, 4987 European as replication sample	
Retinal venular tortuosity	GCST90270399			
Retinal arteriolar width	GCST90270398			
Retinal venular width	GCST90270400			
Type 1 diabetes	GCST90014023	2021	520580	59999551
Type 2 diabetes	GCST010118	2020	433540	11222507
Cardiovascular diseases	GCST90038595	2021	484598	9587836
Stroke	GCST005838	2018	446696	7633440
Ischemic stroke	GCST006908	2018	440328	7537579
Ischemic stroke (cardioembolic)	GCST006910	2018	211763	8271294
Ischemic stroke (small-vessel)	GCST006909	2018	198048	8280845
Ischemic stroke (large artery atherosclerosis)	GCST005840	2018	150765	7992739
Hypertension	GCST90038604	2021	484598	9587836
Diastolic blood pressure	GCST90025981	2021	422713	4228468
Systolic blood pressure	GCST90025968	2021	422713	4228468

### 2.3 Instrumental variable selection

To investigate causal effects using genetic variations, three principal assumptions of IVs must be satisfied as described above (Fig. 1). We utilized the following criteria to obtain qualified IVs. We set a GWAS threshold based on a significance level of *P*<5 × 10^-6^ to ensure that only SNPs strongly associated with the exposures/mediators/outcomes were included, and an adequate number of SNPs were considered as well. Then, we utilized a chained unbalanced aggregation method (Linkage disequilibrium (LD) threshold, *r*^2^<0.001, clumping distance >10000 kb, using 1000 Genomes Project European sample data) to minimize the likelihood of selecting SNPs that are in LD with each other, which would otherwise jeopardize the independence of SNPs representing an independent source of genetic variation. Next, we selected SNPs with effect allele frequencies >0.01 and excluded SNPs with *F*-statistics <10 to ensure the robustness of selected SNPs as IVs. F-statistics were calculated using formula *F*=*beta*^2^/*se*^2^.

### 2.4 Statistical analyses

#### 2.4.1 Bidirectional two-sample Mendelian Randomization

First, we applied a conventional UVMR to evaluate the causal relationships among primary glaucoma and retinal vascular occlusions. We used the Wald ratio to infer causality for specific exposures with only one IV. For exposures comprising multiple IVs, inverse variance-weighted (IVW), MR-Egger, weighted median, simple mode, and weighted mode methods were employed to assess causality, with IVW serving as the primary analysis. Briefly, IVW meta-analyzed SNP-specific Wald estimates using random effects to reach a final estimate of causal effects. The reverse MR methods were similar to forward MR, as described above, with the only difference being the reversal of exposures and outcomes.

#### 2.4.2 Sensitivity analysis

Sensitivity analysis was conducted to assess the robustness of the causal relationships. To evaluate directional pleiotropy, we examined the intercept value of the MR-Egger regression. A non-zero regression intercept with *P* values<0.05 may be considered a statistically significant indicator of genetic pleiotropy. Heterogeneity was assessed using Cochran's *Q* test, with smaller *P*-values indicating higher heterogeneity and a potential for directional pleiotropy. In the absence of heterogeneity, a fixed-effect model was used. Additionally, leave-one-out analysis was used to detect SNP outliers.

#### 2.4.3 Mediation analysis

After conducting a bidirectional two-sample UVMR analysis among exposures, mediators, and outcomes, we performed a mediation analysis using 2-step MR to investigate whether specific mediators mediate causal pathways between primary glaucoma and retinal vascular occlusions. The overall effect can be decomposed into indirect (through a medium) and direct impact (without a medium). Specifically, this includes MR randomization analysis of the effects of the exposure on the mediator (*β*_1_), MR randomization analysis of the effect of the mediator on the outcome (*β*_2_), and calculation of the mediating effect (*β*_1_ × *β*_2_) and direct effect (total effect-mediating effect), as shown in Fig. 1.

### 2.5 Software

All analyses were conducted in the R Studio environment, utilizing R version 4.4.1. We employed the R packages "TwoSampleMR", "ieugwasr", "dplyr", and "vroom" for the efficient reading of Finngen data.

## 3 Results

### 3.1 Causal relationships between primary glaucoma and retinal vascular occlusion

**Fig. 2:**
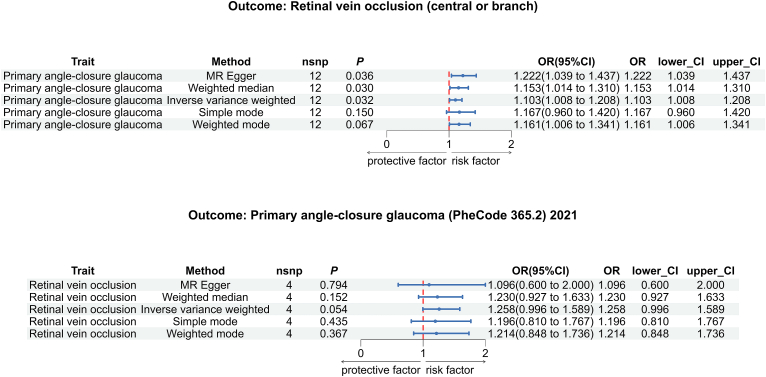
Mendelian Randomization Analysis of Bidirectional Causal Relationships Between Primary Glaucoma and Retinal Vascular Occlusions. This figure shows the MR estimates (OR and 95% CI) for the associations between primary glaucoma (POAG and PACG) and RVO using different MR methods. The top panel indicates a significant causal effect of POAG on RVO, while the bottom panel shows a borderline effect from RVO to PACG.

This study identified 12/9 SNPs representing POAG/PACG and 4/4/4 SNPs representing RVO/BRAO/CRAO as IVs (detailed SNP information see Supplementary Table S1). *F*-statistics of all SNPs are greater than 10, which indicates an absence of weak IV bias. The influences of primary glaucoma on retinal vascular occlusion risk were assessed primarily by the IVW method. After multiple tests for correction and robustness, statistically significant associations were observed between POAG and RVO (*P*=0.032, Supplementary Table S2, Fig. 2), with a one-unit increase in POAG risk corresponding to a 1.103-fold (95% confidence intervals (CI): 1.008-1.206) increased risk of RVO (Supplementary Table S2, Fig. 2). No nominally significant association was found in other forward MR analyses from primary glaucomas to retinal vascular occlusions (Supplementary Table S2).

The reverse MR verified the direction of the genetically causal relationship from POAG to RVO (odds ratio (OR)=0.804, 95% CI: 0.604-1.069, *P*=0.133, Supplementary Table S2). Meanwhile, we observed a positive but statistically non-significant association from genetically predicted RVO (central or branch) to PACG (OR=1.258, 95% CI: 0.996-1.589, *P*=0.054, Supplementary Table S2, Fig. 2), suggesting a need for further investigation into the magnitude of this effect.

### 3.2 MR sensitivity analysis

**Fig. 3:**
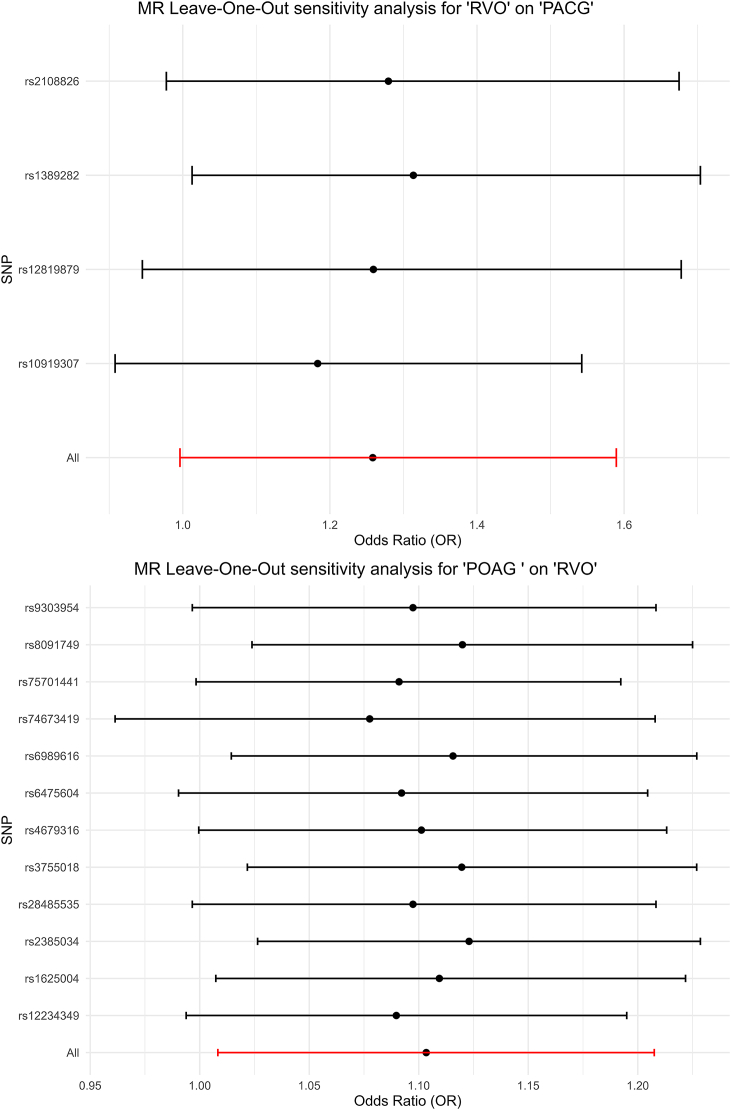
Leave-one-out sensitivity analysis for POAG-RVO and RVO-PACG associations. This figure displays the results of leave-one-out sensitivity analyses conducted using the (IVW method. Each point represents the estimated causal effect after excluding one SNP at a time, evaluating the robustness of the associations between POAG and RVO, and between RVO and PACG.

The sensitivity results for POAG-RVO and RVO-PACG links did not exhibit any sign of heterogeneity according to Cochran's *Q* heterogeneity test (Supplementary Table S3). Additionally, no evidence of directional pleiotropy was found, since the intercepts from the MR-Egger analyses did not deviate from zero (Supplementary Table S3). The leave-one-SNP-out analysis of the IVW estimate for the POAG-RVO and RVO-PACG associations further validated the data robustness from each single SNP (Supplementary Table S4 & Fig. 3).

### 3.3 Mediation analysis

**Fig. 4:**
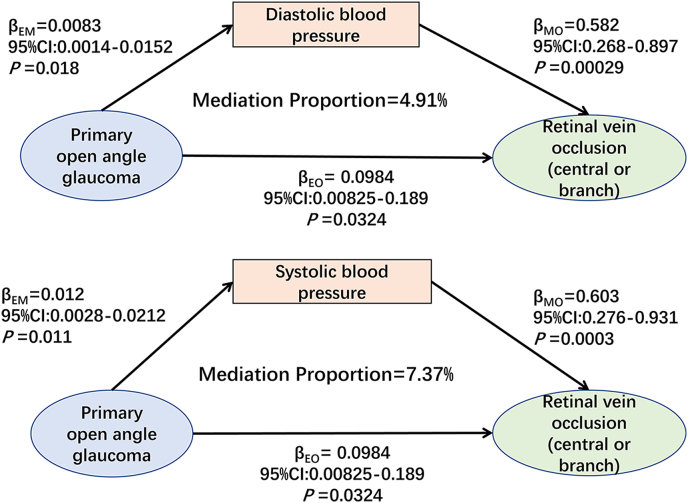
Two-step Mendelian randomization mediation analysis of the causal pathway from POAG to RVO via.BP This figure illustrates the two-step Mendelian randomization mediation analysis assessing whether systolic and diastolic BP mediate the causal effect of POAG on RVO. The diagram presents the estimated causal effects (*β*) and corresponding *P*-values along each path, with the proportion of the mediated effect indicated.

We conducted a Two-Step Mediation analysis of the causal link between POAG-RVO and the latent RVO-PACG relationship. First, we filtered mediators using the standard two-sample MR method as described above, under which circumstances both POAG and RVO act as exposures (*β*_1_) (Supplementary Table S5). Next, comprehensive sensitivity tests and a reverse MR (Supplementary Tables S6 and S7) were also carried out to verify robustness and direction. Then, the causal relationships between mediators and RVO/PACG (*β*_2_) were assessed using the same schedule (Supplementary Tables S8–10). Finally, we received potential mediators holding causal and directional relationships with both exposures (POAG/RVO) and outcomes (RVO/PACG) for the mediation analysis, and the mediated MR analysis showed that systolic and diastolic BP mediated POAG-RVO link, with a mediation proportion of 7.4% (*β*=0.00726, 95% CI: 0.00044-0.014, *P*=0.036) and 4.9% (*β*=0.00483, 95% CI: 0.00005-0.0096, *P*=0.047), respectively (Fig. 4).

## 4 Discussion

The etiological interplay between primary glaucoma and retinal vascular occlusion is a long-standing, controversial topic. Using the bidirectional MR approach, the present study first attempted to investigate the 'The chicken or the egg-which comes first?' question through genetic inference, complementing the current literature. We provide novel genetic evidence supporting a unidirectional causal effect of POAG on the risk of RVO. More importantly, our mediation analysis unveils that systemic BP, encompassing both systolic and diastolic components, acts as a potential mediating pathway in this relationship. This crucial insight extends beyond mere genetic correlation and provides a plausible mechanistic link connecting glaucomatous pathology and retinal vascular events. Furthermore, a latent genetic causality was also observed between RVO and an elevated risk of PACG.

### 4.1 From POAG to RVO (central or branch)

The question of "Which comes first" between primary glaucoma and retinal vascular occlusion has been discussed over the past decades.^1^^,^^2^ First reported and postulated by Verhoeff in 1913 ^3^, elevated IOP, one common characteristic in most types of glaucoma, may be the critical factor responsible for compressing and collapsing the retinal vascular wall, thereby leading to intimal hyperplasia, which eventually leads to blood stasis and vascular occlusion pathogenesis. Thenceforth, numerous studies have investigated the relationship between types of primary glaucoma and retinal vascular occlusions, specifically the relationship between POAG and RVO. However, the POAG-RVO relationship is often challenged after adjusting for aging, a common risk factor for both POAG and RVO.^19^ Here, we provide a robust genetic prediction supporting a directional relationship for the first time. Moreover, we revealed that both systolic and diastolic BP mediated the POAG-RVO link in the present MR study, which is highly congruent with a growing body of pathophysiological evidence.

The structural sequelae of POAG inevitably contribute to this predisposition. The enlarged C/D in POAG postulates vascular structural abnormalities at the optic nerve area induced by optic disc cupping,^20^ and posterior bowing of the lamina cribrosa tissue in POAG confers less glial support to retinal vessels.^21^ POAG patients may have narrower retinal arteries and veins, predisposing them to higher vessel wall compression when encountering an IOP increase.^22^ Compared to artery occlusions, the pathophysiology and risk factors associated with RVO are more vulnerable to ocular changes, such as pressure gradient variations.^23^ Even a slight increase in IOP may be significant enough to jeopardize ocular perfusion, leading to subsequent venous slowing or stasis53.^3^ Therefore, elevated IOP and excessive IOP fluctuation in POAG may further interact with systemic hemodynamic disturbances, exerting a detrimental impact on RVO pathogenesis.^24^ Together, according to Virchow's triad, retinal veins are more sensitive to venous stasis, thrombosis, and eventually occlusion in a POAG condition characterized by excessive IOP increase or fluctuation, as well as glaucomatous structural abnormalities in the optic nerve area.^21^

Both systolic and diastolic BP are well-documented risk factors for RVO^25^, ^26^, ^27^. RVO patients also have abnormal systemic hemodynamic appearance, such as an unfavorable nocturnal hypertensive profile,^28^ early-stage hypertension,^29^^,^^30^ and evidence of atherosclerotic disease. POAG patients may have higher risks of systemic hemodynamic disturbances such as dysregulated variability of diurnal arterial pressure, exaggerated nocturnal BP reduction^31^, ^32^, ^33^, and arterial stiffness.^34^ Emerging data indicate that autonomic nervous system (ANS) dysregulation is a common feature in POAG, characterized by decreased heart rate variability (reflecting impaired adaptability),^35^^,^^36^ excessive sympathetic activation, and reduced parasympathetic tone.^35^, ^36^, ^37^, ^38^ This autonomic imbalance serves as a key physiological link. Within the eye, sympathetic overactivity can induce microvascular dysregulation in the choroid and optic nerve head, where vasoconstriction compromises blood flow and exacerbates glaucomatous damage.^37^, ^38^, ^39^, ^40^ Systemically, the same autonomic dysfunction manifests as hemodynamic instability and BP fluctuations,^41^ creating a hostile circulatory environment. The structural vulnerability of POAG may intersect with ANS-driven systemic BP instability—particularly episodes of hypotension or excessive fluctuation—the retinal venous circulation faces a dual threat: reduced perfusion pressure and mechanical compression. This scenario perfectly aligns with Virchow's triad, where BP instability contributes to stasis, and glaucomatous changes predispose to endothelial stress, collectively fostering a pro-thrombotic state that culminates in RVO.

Beyond innate pathophysiology, our findings raise essential considerations regarding iatrogenic and psychological influences. A substantial proportion of patients with POAG are treated with topical β-adrenergic blockers. While effective in lowering IOP, their systemic absorption can lead to clinically significant reductions in heart rate and BP, potentially disrupting the circadian rhythm of BP and resulting in nocturnal hypotension^42^, ^43^, ^44^, ^45^, ^46^, ^47^, ^48^. This pharmacologic effect may unintentionally exacerbate the very hemodynamic instability that our study implicates in the pathogenesis of RVO. Furthermore, the chronic psychological stress associated with a progressive, sight-threatening condition like POAG cannot be overlooked. Chronic stress activates the hypothalamic-pituitary-adrenal axis and the ANS, which can elevate IOP and disturb BP homeostasis.^49^ If POAG contributes to chronic stress, a self-reinforcing feedback loop may be established, wherein the disease state perpetuates the neuroendocrine and hemodynamic disturbances that indirectly promote RVO. Recent epidemiological work by Umetsu et al. (2024) further supports a potential causal role of ocular hypertension in promoting systemic hypertension,^50^ possibly through mechanisms involving the renin-angiotensin system and oxidative stress.^51^^,^^52^ Collectively, these lines of evidence paint a picture of a complex, multidirectional interplay where POAG, through a combination of autonomic dysregulation, structural compromise, pharmacological side effects, and stress responses, creates a systemic milieu conducive to vascular occlusion.

### 4.2 From RVO (central or branch) to PACG

Compared to the POAG-RVO link, existing literature on the relationship between RVO and PACG is modestly scarce, which limits the power to conclude meaningful and reliable associations between PACG and RVO risk.^1^ According to a recent meta-analysis,^2^ PACG was associated with central retinal vein occlusion (CRVO) (OR: 5.3, 95% CI: 1.04–26.95, *P*=0.045), but not with branch retinal vein occlusion (BRVO) (OR: 0.65, 95% CI: 0.07–6.27, *P*=0.707). However, the small number of studies with limited sample sizes enrolled in that meta-analysis confined the reliability of such a conclusion.^2^ Some researchers hold the plausible opinion that PACG may exert detrimental impacts on RVO, similarly to POAG, through the mechanical displacement of the central venous trunk due to optic cupping and stretching towards the vein wall.^2^ Nevertheless, we must be aware that the pathogenesis of PACG and POAG is quite different, and the retinal vasculature parameters differ significantly between PACG and POAG.^53^

We observed a latent connection between RVO and PACG in this MR study, with a borderline significance of *P*=0.054. Vascular congestion and edema in the posterior tissue of the RVO condition may lead to rotation of the anterior ciliary diaphragm, thereby narrowing the anterior drainage angle.^54^^,^^55^ Moreover, before the preclinical stage of PACG, primary angle closure (PAC) is reported to be more prevalent in RVO and its fellow eyes as well.^56^^,^^57^ Therefore, despite the majority of existing literature proposing pre-existing PACG/PAC as a well-characterized risk factor for RVO,^55^ we still hold a humble opinion that more data from high-quality epidemiologic studies focusing on RVO-PACG/PAC relationships, particularly with fellow eyes investigation, are needed in the future to verify our genetic prediction and to confirm this etiological connection.

### 4.3 Strengths and limitations

Our study has several strengths that warrant highlighting. First, this study represents the first large-scale genetic investigation of the causal relationships between different types of primary glaucoma and retinal vascular occlusions. Second, our MR approach uses genetic variables as a research tool to mitigate biases inherent in observational studies. We assessed participants of European ancestry to reduce the risk of population stratification. Third, this MR study features a large sample size, ensuring robust estimation effects for each instrumental variable, with *F-*statistics exceeding 10, thereby effectively safeguarding the statistical power of this study. Finally, our findings were supported by the execution of various sensitivity analyses. Cochrane's *Q* and MR-Egger intercept tests were employed to assess further and mitigate pleiotropy.

Meanwhile, several limitations shall also be acknowledged. First, IV selection was based on the threshold of *P*<5 × 10^-^^6^, since we could not acquire adequate SNPs at the threshold of *P*<5 × 10^-^^8^ nor *P*<1 × 10^-^^6^ in some circumstances. To address this altered threshold, comprehensive sensitivity analysis and robustness checks were added. Second, we acknowledge that restricting our analysis to European-ancestry populations limits the generalizability of our findings. This methodological choice was crucial in minimizing bias from population stratification, a known source of spurious associations in genetic studies. However, we recognize that the genetic architecture of both primary glaucoma and retinal vascular occlusion may differ across ethnicities. The current scarcity of large-scale, well-powered GWAS summary statistics for these specific traits in non-European populations remains a significant barrier to conducting trans-ethnic Mendelian randomization analyses. We strongly advocate for and anticipate that future efforts to build diverse genetic resources will be crucial for validating and translating these causal insights across different ancestry groups. Third, different subtypes of RVO, such as BRVO, CRVO, and hemiretinal vein occlusion (HRVO), may have various pathogeneses, and different RVO subtypes may respond differently to glaucomatous impacts.^58^ However, since MR studies are secondary analyses relying on existing GWAS data, we were unable to find more detailed data differentiating subtypes of RVO in Finngen, which only included a general classification of RVO (central or branch). This should be noted in the interpretation of our results. Fourth, our study is subject to limitations inherent to the source data. Despite utilizing the UK Biobank, a widely recognized and authoritative database, the number of cases identified through strict ICD-10 coding for the primary glaucoma exposure was relatively limited, which can be considered a constraint that might affect the robustness of the genetic instruments and the subsequent MR estimates. Therefore, our results should be interpreted with caution and await validation in future studies with larger case cohorts. Finally, despite the use of various methods to mitigate the influence of pleiotropy, the potential bias of unknown pleiotropy on the results cannot be entirely dismissed.

## 5 Conclusions

In conclusion, this MR study suggests an increased risk of RVO associated with POAG and a potential connection between RVO and PACG. According to additional mediation analysis, we suggest that the partial effect of POAG on RVO risk may be mediated through systolic and diastolic BP. These findings highlight an intricate interplay among POAG, RVO, and systemic hemodynamics, providing new avenues for further exploration of mechanisms and future clinical practice.

## Study approval

Not Appliable.

## Author contributions

The authors confirm contribution to the paper as follows: Conceptualization, Y.Z., and Y.C.; methodology & software, J.L.; validation & formal analysis, C.W.; investigation, T.Z.; data curation, Xinyi.L.; writing—original draft preparation, Y.Z., C.W., K.T.; supervision, W.S., X.Y., first major revision, Xinmin.L.; second minor revision, Y.Y., C.W., T.Z., and Y.Y. contributed equally to this research and should be considered as equivalent authors. J.L., Y.C., Xinmin.L., and Y.Z. should be considered as corresponding authors. All authors have read and agreed to the current version of the manuscript.

## Data availability statement

The summary statistics from the UK BioBbank are available at https://pan.ukbb.broadinstitute.org (last access date: 2024/8). The retinal vascular occlusion summary statistics from the FinnGen are available at https://www.finngen.fi/en/access_results (last access date: 2024/8). Other datasets presented in this study can be found in online repositories at https://gwas.mrcieu.ac.uk/and https://gwas.mrcieu.ac.uk/and https://www.ebi.ac.uk/gwas/downloads/summary-statistics. Accession number(s) can be found in Table 1.

## Funding

This work was supported by College-level Project Fund of Shanghai Sixth People's Hospital Affiliated to Shanghai Jiao Tong University School of Medicine (Grant No. ynts202213), National Natural Science Foundation of China (82501245), China Postdoctoral Science Foundation (2025M771943), and Shanghai Postdoctoral Excellence Program (2024409).

## Declaration of competing interest

The authors declare that they have no known competing financial interests or personal relationships that could have appeared to influence the work reported in this paper.

## Abbreviations

POAG: Primary open-angle glaucoma
PACG: Primary angle-closure glaucoma
RVO: Retinal vein occlusion
RAO: Retinal artery occlusion
IOP: Intraocular pressure
RNFL: Retinal nerve fiber layer
GCIPL: Ganglion cell inner plexiform layer
BRAO: Branch retinal artery occlusion
CRAO: Central retinal artery occlusion

## References

[1] Jabbehdari S., Yazdanpanah G., Cantor L.B. (2022). A narrative review on the association of high intraocular pressure and glaucoma in patients with retinal vein occlusion. Ann Transl Med.

[2] Yin X., Li J., Zhang B. (2019). Association of glaucoma with risk of retinal vein occlusion: a meta-analysis. Acta Ophthalmol (Copenh).

[3] Verhoeff F. (1913). The effect of chronic glaucoma on the central retinal vessels. Arch Ophthalmol.

[4] Bhoot M., Pegu J., Bhumbla S. (2022). Association of primary angle-closure disease in patients with retinal vein occlusion in north Indian population. Indian J Ophthalmol.

[5] Călugăru M. (2000). The incidence of primary open-angle glaucoma in patients with central retinal vein occlusion. Oftalmol (Buchar Rom: 1990).

[6] Sherpa D., Shakya S., Shrestha J.K. (2008). Association of primary glaucomas with retinal vein occlusion. Kathmandu Univ Med J (KUMJ).

[7] Ørskov M., Vorum H., Larsen T.B. (2022). Clinical risk factors for retinal artery occlusions: a nationwide case-control study. Int Ophthalmol.

[8] Hitchings R.A., Spaeth G.L. (1976). Chronic retinal vein occlusion in glaucoma. Br J Ophthalmol.

[9] Vannas S., Tarkkanen A. (1960). Retinal vein occlusion and glaucoma. Tonographic study of the incidence of glaucoma and of its prognostic significance. Br J Ophthalmol.

[10] Lindblom B. (1998). Open angle glaucoma and non-central retinal vein occlusion--the chicken or the egg?. J Acta Ophthalmol Scand.

[11] De Salvo G., Oshallah M., Sepetis A.E. (2023). Inner retinal thinning comparison between branch retinal artery occlusion and primary open-angle glaucoma. J Diagn (Basel Switz).

[12] Melgarejo J.D., Van Eijgen J., Wei D. (2023). Effect of 24-h blood pressure dysregulations and reduced ocular perfusion pressure in open-angle glaucoma progression. J Hypertens.

[13] Dinakaran S., Mehta P., Mehta R. (2022). Significance of non-intraocular pressure (IOP)-related factors particularly in normal tension glaucoma: looking beyond IOP. Indian J Ophthalmol.

[14] Raman P., Suliman N.B., Zahari M. (2018). Low nocturnal diastolic ocular perfusion pressure as a risk factor for NTG progression: a 5-year prospective study. Eye (Lond Engl).

[15] Quaranta L., Katsanos A., Riva I. (2016). Twenty-four-hour intraocular pressure and ocular perfusion pressure characteristics in newly diagnosed patients with normal tension glaucoma. Eye (Lond Engl).

[16] Shin Y.I., Nam K.Y., Lee S.E. (2019). Changes in peripapillary microvasculature and retinal thickness in the fellow eyes of patients with unilateral retinal vein occlusion: an OCTA study. Investig Ophthalmol Vis Sci.

[17] Skrivankova V.W., Richmond R.C., Woolf B.A.R. (2021). Strengthening the reporting of observational studies in epidemiology using mendelian randomization: the STROBE-MR statement. JAMA.

[18] Lawlor D.A., Harbord R.M., Sterne J.A.C. (2008). Mendelian randomization: using genes as instruments for making causal inferences in epidemiology. Stat Med.

[19] Klein R., Klein B.E., Moss S.E. (2000). The epidemiology of retinal vein occlusion: the beaver dam eye study. Trans Am Ophthalmol Soc.

[20] Beaumont P.E., Kang H.K. (2002). Cup-to-disc ratio, intraocular pressure, and primary open-angle glaucoma in retinal venous occlusion. Ophthalmology.

[21] Behrman S. (1962). Retinal vein obstruction. Br J Ophthalmol.

[22] Sonnsjö B., Krakau C.E. (1993). Arguments for a vascular glaucoma etiology. Acta Ophthalmol (Copenh).

[23] Ørskov M., Vorum H., Larsen T.B. (2023). Similarities and differences in systemic risk factors for retinal artery occlusion and retinal vein occlusion: a nationwide case-control study. Int Ophthalmol.

[24] Nenciu A., Stefan C., Tebeanu E. (2005). Retinal venous occlusion and intraocular pressure. Oftalmol (Buchar Rom: 1990).

[25] Arakawa S., Yasuda M., Nagata M. (2011). Nine-year incidence and risk factors for retinal vein occlusion in a general Japanese population: the hisayama study. Investig Ophthalmol Vis Sci.

[26] Kalva P., Akram R., Zuberi H.Z. (2023). Prevalence and risk factors of retinal vein occlusion in the United States: the national health and nutrition examination survey, 2005 to 2008. Proc (Bayl Univ, Med Cent).

[27] Laouri M., Chen E., Looman M. (2011). The burden of disease of retinal vein occlusion: review of the literature. Eye (Lond Engl).

[28] García-Tellado Á., Solís-Sánchez P., Cerveró A. (2023). Evaluation of the ambulatory blood pressure monitoring in patients with retinal vein occlusion. Med Clin (Barc).

[29] Hashimoto Y., Kaneko H., Okada A. (2022). Association between retinal vein occlusion and life's simple 7 cardiovascular health metrics: a large claims database study. Ophthalmol, Retina.

[30] Hashimoto Y., Kaneko H., Aso S. (2023). Association between retinal vein occlusion and early-stage hypertension: a propensity score analysis using a large claims database. Eye (Lond Engl).

[31] Melgarejo J.D., Eijgen J.V., Wei D. (2022). Progression of functional and structural glaucomatous damage in relation to diurnal and nocturnal dips in mean arterial pressure. Front Cardiovasc Med.

[32] Marjanović I., Marjanović M., Martinez A. (2016). Relationship between blood pressure and retrobulbar blood flow in dipper and nondipper primary open-angle glaucoma patients. Eur J Ophthalmol.

[33] Pillunat K.R., Spoerl E., Jasper C. (2015). Nocturnal blood pressure in primary open-angle glaucoma. Acta Ophthalmol (Copenh).

[34] Visontai Z., Mersich B., Holló G. (2005). Carotid artery elasticity and baroreflex sensitivity in patients with glaucoma. J Glaucoma.

[35] Na K.S., Lee N.Y., Park S.H. (2010). Autonomic dysfunction in normal tension glaucoma: the short-term heart rate variability analysis. J Glaucoma.

[36] Park H.Y.L., Park S.H., Park C.K. (2014). Central visual field progression in normal-tension glaucoma patients with autonomic dysfunction. Investig Ophthalmol Vis Sci.

[37] Reiner A., Fitzgerald M.E.C., Del Mar N. (2018). Neural control of choroidal blood flow. Prog Retin Eye Res.

[38] Flammer J., Orgül S., Costa V.P. (2002). The impact of ocular blood flow in glaucoma. Prog Retin Eye Res.

[39] Flammer J., Mozaffarieh M. (2008). Autoregulation, a balancing act between supply and demand. J Can Ophtalmol.

[40] Wierzbowska J., Wierzbowski R., Stankiewicz A. (2012). Cardiac autonomic dysfunction in patients with normal tension glaucoma: 24-h heart rate and blood pressure variability analysis. Br J Ophthalmol.

[41] Ramdas W.D., Wolfs R.C.W., Hofman A. (2011). Ocular perfusion pressure and the incidence of glaucoma: real effect or artifact? The rotterdam study. Investig Ophthalmol Vis Sci.

[42] Nelson W.P. (1987). Adverse respiratory and cardiovascular events attributed to timolol ophthalmic solution, 1978-1985. Am J Ophthalmol.

[43] Fraunfelder F.T., Meyer S.M. (1989). Systemic adverse reactions to glaucoma medications. Int Ophthalmol Clin.

[44] Atkins J.M., Pugh B.R., Timewell R.M. (1985). Cardiovascular effects of topical beta-blockers during exercise. Am J Ophthalmol.

[45] Monane M., Bohn R.L., Gurwitz J.H. (1994). Topical glaucoma medications and cardiovascular risk in the elderly. Clin Pharmacol Ther.

[46] Hayreh S.S., Zimmerman M.B., Podhajsky P. (1994). Nocturnal arterial hypotension and its role in optic nerve head and ocular ischemic disorders. Am J Ophthalmol.

[47] Netland P.A. (2000). Beta-blocker eyedrops and nocturnal arterial hypotension. Am J Ophthalmol.

[48] Graham S.L., Drance S.M. (1999). Nocturnal hypotension: role in glaucoma progression. Surv Ophthalmol.

[49] Won E., Kim Y.K. (2016). Stress, the autonomic nervous system, and the immune-kynurenine pathway in the etiology of depression. Curr Neuropharmacol.

[50] Umetsu A., Tanaka M., Sato T. (2024). High intraocular pressure is independently associated with new-onset systemic hypertension over a 10-year periodJ. Circ J: Off J Jpn Circ Soc.

[51] Yasukawa T., Hanyuda A., Yamagishi K. (2022). Relationship between blood pressure and intraocular pressure in the JPHC-NEXT eye study. Sci Rep.

[52] Mogi M., Ikegawa Y. (2025). Close relationship between systemic blood pressure and intraocular pressure. Hypertens Res: Off J Jpn Soc Hypertens.

[53] Cheng C.S.M., Lee Y.F., Ong C. (2016). Inter-eye comparison of retinal oximetry and vessel caliber between eyes with asymmetrical glaucoma severity in different glaucoma subtypes. Clin Ophthalmol (Auckl NZ).

[54] Grant W.M. (1973). Shallowing of the anterior chamber following occlusion of the central retinal vein. Am J Ophthalmol.

[55] Wu S.C., Lee Y.S., Wu W.C. (2016). Anterior chamber depth and angle-closure glaucoma after central retinal vein occlusion. BMC Ophthalmol.

[56] Jonas J.B., Nangia V., Khare A. (2013). Prevalence and associations of retinal vein occlusions: the central India eye and medical study. Retina (Phila Pa,).

[57] Xu K., Wu L., Ma Z. (2019). Primary angle closure and primary angle closure glaucoma in retinal vein occlusion. Acta Ophthalmol (Copenh).

[58] Beaumont P.E., Kang H.K. (2002). Clinical characteristics of retinal venous occlusions occurring at different sites. Br J Ophthalmol.

